# Unexplained anemia: A case report of metachronous adenocarcinoma arising in the transverse colon following right hemicolectomy for a primary cecal carcinoma

**DOI:** 10.5339/qmj.2021.16

**Published:** 2021-04-30

**Authors:** Asim Malik, -Ur-Rahman Asad, Syed Muhammad Hammad Ali

**Affiliations:** ^1^Department of Surgery, FMH College of Medicine & Dentistry, Lahore, Pakistan E-mail: hali921@hotmail.com; ^2^Digestive Disease Institute, Cleveland Clinic, Florida, USA

**Keywords:** Metachronous, colorectal, adenocarcinoma, anemia, gastrointestinal (GI) bleeding, hemicolectomy

## Abstract

Metachronous colonic carcinomas arise from months to years after the resection of the first or index primary colorectal cancer. They are not a result of tumor recurrence or metastasis and likely arise as a result of the field cancerization effect. This report presents the case of a 63-year-old male patient without family history of a colorectal cancer but had an index primary adenocarcinoma of the cecum (stage IIIC) five years ago that was treated with surgical resection and adjuvant radiotherapy and chemotherapy. He presented with fatigue and anemia of 6-month duration secondary to recurrent melena, and the specific cause of which remained obscure despite intensive diagnostic workup. Recurrence of a malignancy at the previous anastomosis site was ruled out. The patient continued to have recurrent and intermittent gastrointestinal bleeding until a nuclear red blood cell scan detected a bleeding spot in the epigastric region, which actually turned out to be a second primary carcinoma (stage I) arising from an adenoma in the transverse colon. The patient underwent a left colectomy with ileosigmoid anastomosis formation. During a two-month postoperative follow-up, the patient did not experience any episode of melena or anemia. Even though metachronous colon cancers rarely present with a recurrent and intermittent gastrointestinal bleeding with melena, an aggressive workup must be aimed at ruling out a second independent malignancy in patients who are in remission after an index primary colorectal cancer resection through hemicolectomy. Any neoteric lesion found on colonoscopy in such cases should be dealt with a higher degree of suspicion. Therefore, the need for surveillance colonoscopy as recommended by the National Comprehensive Cancer Network guidelines is imperative and should be practiced in resource-limited countries.

## Introduction

Multiple primary carcinomas are defined as the presence of more than one primary cancer in a single patient. Index cancer is the earliest to arise, which may be followed by other independent primary cancers in the same or other organs. However, these must not be a result of recurrence, contiguous spread, or distant metastasis and are diagnosed on histology.^1^ Metachronous colorectal carcinomas can present years after resection of the index primary cancer and occur rarely, with incidence rates varying from 2% to 20%.^2,3^ Herein, we present a case of anemia due to an obscure gastrointestinal (GI) bleeding, which subsequently developed into a metachronous adenocarcinoma of the transverse colon, emerging almost 5 years after the surgical resection of the index primary carcinoma of the cecum.

## Case Presentation

A 63-year-old male patient presented to our clinic with weakness and recurrent melena for the last six months. His past history was significant for a stage IIIC (T4a, N2a, M0) adenocarcinoma of the cecum, which was diagnosed and treated five years back. In December 2014, he underwent an extended right hemicolectomy with ileocolic anastomosis formation, followed by radiation and chemotherapy. The patient was deemed to be in remission after follow-up 18F-fluorodeoxyglucose-positron emission tomography (FDG-PET) scans did not show any sign of a residual disease. He lost colonoscopy follow-up after one year of disease remission. His other comorbidities included a poorly controlled type II diabetes mellitus and hypertension. He had a surgical history of coronary artery bypass grafting and a cholecystectomy. His family history was negative for colorectal carcinomas.

The patient was doing all well until six months ago when he started noticing intermittent blackening of his stools while experiencing unusual fatigue. He sought medical attention and was admitted to a tertiary care hospital before presenting to us. His initial laboratory workup showed a hemoglobin (Hb) level of 7 g/dl with stool positive for occult blood. Ultrasonography and computed tomography (CT) scans of the abdomen and pelvis were unremarkable. As a part of the diagnostic workup, he underwent a total of four esophagogastroduodenoscopies (EGD) and three colonoscopies at various intervals during the next six months to rule out the cause for his intermittent GI bleeding. EGDs showed some nonspecific findings of mild erosive esophagitis/gastritis, while colonoscopies revealed several aphthous ulcers and ectasias of the large intestine as well as fresh blood in the entire colon and terminal ileum. Argon plasma coagulation therapy was performed at the sites of angioectasia. There was no evidence of tumor recurrence at the previous ileocolic anastomosis site, and the concurrent carcinoembryonic antigen (CEA) levels were also normal. However, an unusual polypoid lesion with oozing blood was also seen in the transverse colon. The lesion could not be removed completely because of the wider base; therefore, a biopsy was taken, which showed features of a tubular adenoma. Several biopsies from other sites of the colon showed chronic inflammatory infiltrate in the lamina propria, suggesting radiation colitis as the probable diagnosis.

Despite several colonoscopies and packed red blood cell (RBC) transfusions, the patient continued to have symptoms of intermittent GI bleeding and anemia. He was referred to us for further evaluation of his obscure and intermittent GI bleeding. Baseline investigations revealed an Hb level of 8 g/dl, for which he was given packed cell transfusions. All other chemistry and serological parameters were unremarkable, except for a persistently low white blood cell count (1.70 × 10^3^μl) and a low serum albumin level (2.4 g/dl). Vital signs were within normal limits. Rectal examination showed black, tarry stools.

**Figure 1.**
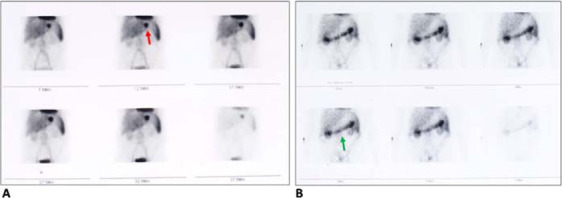
A technetium-99 m (^99m^Tc)-labeled red blood cell (RBC) scintigraphy. A) Imaging during the first 30 minutes of injecting ^99m^Tc-labeled RBCs shows a focal site of active gastrointestinal bleeding near the epigastric and left hypochondriac region (red arrow). B) Delayed imaging performed 18 hours after ^99m^Tc-labeled RBCs injection shows tagged RBCs in the transverse colon (green arrow).

**Figure 2.**
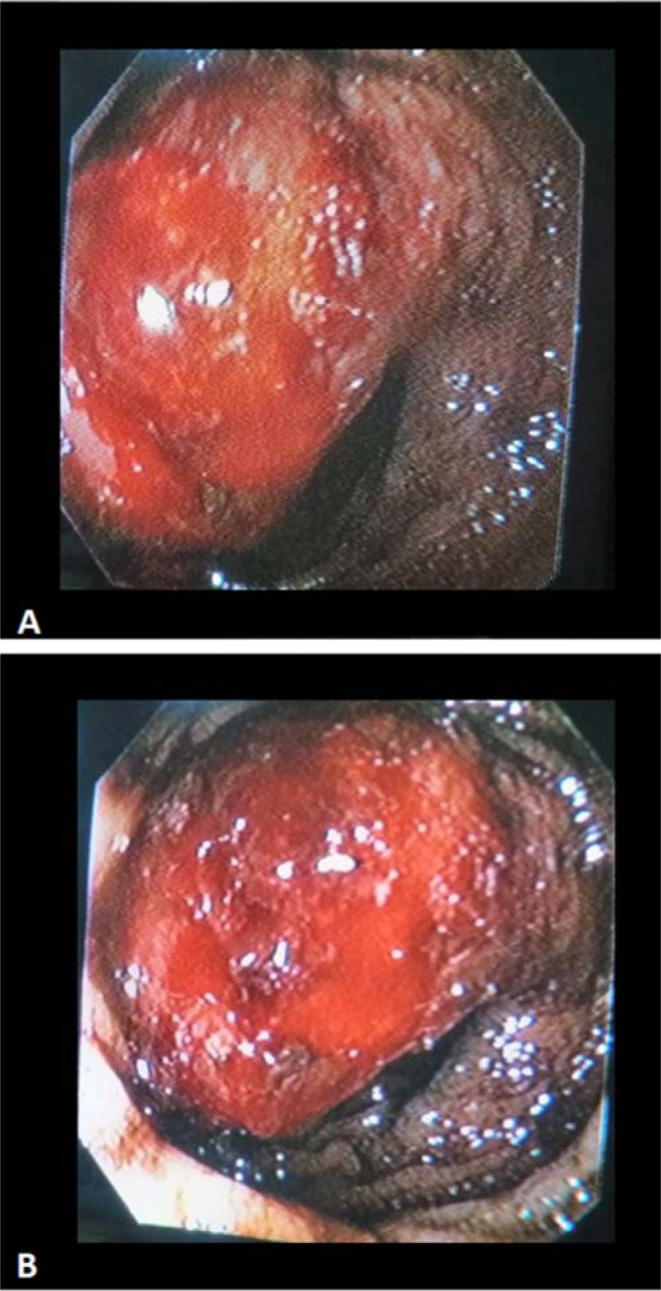
An irregular growth with oozing blood in the transverse colon as seen on colonoscopy.

**Figure 3.**
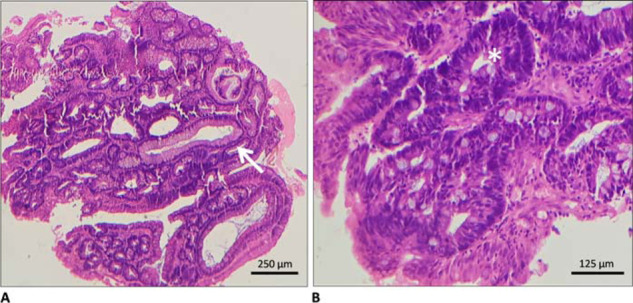
Histopathological appearance of colonoscopic biopsies taken from the growth in the transverse colon on hematoxylin and eosin staining under the light microscope. A) Tubular gland formation consistent with the tubular adenoma (white arrow) (40 × magnification). B) Areas of high-grade dysplasia (asterisk) with hyperchromasia, loss of nuclear polarity, and nuclear stratification (200 × magnification).

A CT angiography was performed, which failed to identify the bleeding vessel. Subsequently, a technetium-99 m (^99m^Tc)-labeled RBC scintigraphy was performed, which revealed a focal site of active GI bleed bordering the epigastric and left hypochondriac region. However, delayed imaging (performed 18 hours later) showed tagged RBCs in the transverse colon, suggesting transit of tracer through the GI tract (Figure 1). Interestingly, concurrent EGD performed to specify the site and nature of lesion came back normal. No ulcer or any lesion corresponding to the gastroduodenal site of bleeding on the nuclear scan was identified, and the mucosal integrity was intact. Colonoscopy was then planned again to meticulously review the previous ileocolic anastomosis site and to rule out tumor recurrence. An irregular growth measuring 2.0 × 2.0 cm^2^ with oozing blood was identified in the transverse colon, approximately 8.0 cm proximal (considering anal verge as the reference point) to the ileocolic anastomosis site (Figure 2). The rest of the colonic mucosa was normal. Histopathology showed features of tubular adenoma with focal areas of high-grade dysplasia (Figure 3).

**Figure 4.**
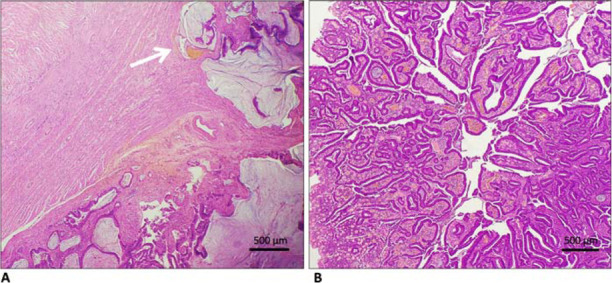
Histopathological appearance of 2.0 × 2.0 × 2.0 cm^3^ polypoid, moderately differentiated adenocarcinoma taken out from the surgically resected transverse colon on hematoxylin and eosin staining under the light microscope (40 ×  magnification). A) Tumor cells invading the muscularis propria (white arrow). B) Adenomatous features showing multiple tubular glands suggestive of tumor arising in the background of a tubular adenoma.

The patient was scheduled for surgery after obtaining informed consent. An exploratory laparotomy was performed along with the left colectomy. The remaining transverse colon as well as its mesentery and the descending colon were removed, and an ileosigmoid anastomosis was formed. The postoperative recovery was uneventful. The histopathology of the 2.0 × 2.0 × 2.0 cm^3^ polypoid tumor confirmed the diagnosis of moderately differentiated adenocarcinoma arising in the background of an adenoma (Figure 4). Two resected regional lymph nodes were also examined, which were free of any metastasis. It was a stage I (T2, N0, M0) disease according to the American Joint Committee of Cancer Staging Classification (Eighth Edition, 2016). The patient did not consent for further genetic mutation testing. During a two-month postoperative follow-up, the patient did not experience any GI bleeding, melena, or anemia. He was readmitted once for dehydration and prerenal azotemia due to infectious diarrhea, for which he was appropriately managed.

Consent was taken from the patient, and approval was sought from the institutional review board prior to writing this case report.

## Discussion

Field cancerization, or field defect, refers to a pattern of neoplastic transformation of a cell population that encompasses a wide "field or patch," where a morphological appearance of a tumor can occur at a particular site, yet the whole field carries some procarcinogenic alterations at the molecular level.^4,5^ Therefore, a second, third, or even more primary malignancies can arise further at a different site within the affected field.

According to the Surveillance Epidemiology and End Results Program, a metachronous lesion is a primary cancer that is not present at the time of diagnosis of an index cancer and emerges separately, after at least two months, in contrast to the synchronous lesions, which are present at the time or within two months of diagnosis of the index cancer.^6,7^ However, the International Agency for Research on Cancer guidelines are more stringent in reporting the incidence of multiple primary carcinomas.^8^ Nevertheless, these lesions should not represent a direct extension, recurrence, or distant metastasis of the index primary cancer, and the diagnosis must be confirmed based on the histological appearance. Risk factors for metachronous colorectal cancer include synchronous cancer or polyps, presence of a metachronous cancer, proximal colon cancer, and hereditary non-polyposis colorectal cancer syndrome.^9^ However, extended surgical resections for index primary carcinomas are generally not recommended unless several factors point toward a risk for development of future malignancy in the patient.^10^ Prognosis largely depends on the stage of the current metachronous disease.

The second primary tumor in our patient emerged nearly five years after resection of the index metastatic carcinoma of the cecum. In a study by Freeman,^11^ all patients with an early stage, non-metastatic colorectal carcinoma developed a second malignancy at least seven years after the diagnosis of the index primary cancer. In our case, the background of an adenoma on histopathology is suggestive of a new cancerous growth independent of the previous tumor, which was also a tubular adenocarcinoma. Furthermore, this patient presented with intermittent GI bleeding, which is quite atypical of a metachronous colorectal adenoma or cancer.^12^ GI bleeding can be tricky to diagnose in some cases, especially with intermittent form. This patient underwent several EGDs and colonoscopies before presenting to us. Therefore, more sensitive tests such as CT angiography and nuclear scintigraphy were performed initially. Scintigraphy imaging with ^99m^Tc-labeled RBCs can detect up to 0.1 mL/min of GI bleeding, and because of the longer half-life of the tracer, delayed imaging allows detection of an intermittent bleeding that can be difficult otherwise.^13,14^ Single-photon emission CT (SPECT) combined with nuclear scintigraphy can further help in localizing the bleeding spot. In our opinion, the delayed imaging (performed 18 hours after injection of ^99m^Tc-labeled RBCs) that picked up the tracer activity from the transverse colon might have been due to an independent bleeding source from the metachronous tumor site that was underlying the gastroduodenal region on SPECT scans.

The most likely differential diagnosis in this patient was tumor recurrence, which was effectively ruled out by colonoscopic evaluation of the previous anastomosis site and normal concurrent CEA levels. CEA levels are often used as a surveillance marker for the recurrence after colorectal cancer resection; however, the sensitivity and specificity is low.^15^ The diagnostic value of serial CEA levels is also controversial. In our case, we did not repeat measurement of the CEA levels after the initial normal results in the laboratory workup. Elevated CEA levels, when combined with FDG-PET scan findings, can be useful in detecting radiologically occult malignancies.^16^ Nonetheless, the role of CEA levels in differentiating tumor recurrence from metachronous lesions remains undocumented. Chronic radiation colitis was also a probable diagnosis, as it can present with GI bleeding and may occur anywhere from three months to 30 years after the exposure to radiation therapy. Colonoscopy findings are nonspecific, but histopathology can show features of hyalinization with fibroblasts, obliterative arteritis, and telangiectasias.^17^ The treatment for radiation colitis is mainly supportive, unless complicated by bleeding, obstruction, and stricture or fistulae formation. However, evaluation to rule out a concurrent malignancy is the most important. Other differential diagnoses could be arteriovenous malformations, neuroendocrine tumors, lymphoma, etc. Park et al. reported an average age of 53 years in patients with metachronous colorectal carcinomas.^18^ By contrast, the second primary malignancy in our patient emerged at age >60 years.

Our patient underwent an extended right hemicolectomy with curative intent for his index primary cecal carcinoma (stage IIIC), since no evidence of any synchronous lesion was present at the time of diagnosis. Adjunct radiotherapy and chemotherapy were planned by the multidisciplinary team to further minimize the risks of tumor recurrence. Likewise, a left colectomy was performed for the metachronous tumor in the transverse colon, but the need for adjunct chemotherapy or radiotherapy was ruled out because of the early stage disease (stage I) and considering his advanced age and comorbidities. Even so, extended colonic resections may result in a poor quality of life and loss of bowel functions and should be reserved for high-risk patients, such as those with Lynch syndrome or with multiple synchronous lesions.^10^

The incidence of metachronous colorectal cancer varies worldwide, ranging from 2% to 20%.^2,3^ This variation is attributed to different levels of disease surveillance. However, given the lack of a comprehensive national cancer registry or surveillance program in Pakistan, epidemiological data of metachronous colorectal carcinomas are not yet available. Moreover, the global incidence of metachronous carcinomas is increasing probably because of improved survival with advanced surveillance, diagnostic techniques, and treatment modalities for the primary colorectal carcinomas.^19^ In developing countries such as Pakistan, factors such as patient’s compliance and affordability pose a huge challenge in maintaining the standard postoperative follow-up. Guidelines suggest postoperative colonoscopy surveillance with an interval of one, three, and five years. However, more intense surveillance can be conducted in patients with specific risk factors, particularly in those with Lynch syndrome and proximal colon cancer.^9^

An important limitation of this case report is the unavailability of genetic mutation testing of the tumor, which is crucial to exclude genetic syndromes. However, there was no history of colon or any other cancer in the patient’s family.

## Conclusion

Patients in remission from colorectal cancer following a hemicolectomy can develop further independent malignancies in the remaining bowel segment. Therefore, patients surviving a colorectal cancer resection must undergo regular colonoscopy surveillance with a higher degree of suspicion for any new lesion formation that is accompanied with an intermittent GI bleeding and melena. Compliance with the recommendations of the National Comprehensive Cancer Network regarding colonoscopic surveillance is also vital in resource-limited countries.

## List Of Abbreviations

CEA, carcinoembryonic antigen; GI, gastrointestinal; FDG-PET, 18F-fluorodeoxyglucose-positron emission tomography; Hb, hemoglobin; CT, computed tomography; EGD, esophagogastroduodenoscopy; ^99m^Tc, technetium-99 m; RBC, red blood cell; SPECT, single-photon emission computed tomography

### Ethical approval

Consent was taken from the patient before writing this case report.

### Conflict of interests

The authors declare that they have no competing interests. No funding was involved in this case report.

### Author’s contributions

Dr. AM was the chief surgeon who performed the surgery on this patient and was involved in the clinical management. Dr. SMHA collected all data relevant to this case. Dr. AM, Dr. AUR, and Dr. SMHA did the literature review. Dr. AUR and Dr. SMHA wrote the introduction and the discussion section. Dr. AM wrote the case presentation. Dr. AM and Dr. AUR reviewed and supervised the whole article. All authors read and approved the final manuscript.

## References

[1] Vogt A, Schmid S, Heinimann K, Frick H, Herrmann C, Cerny T, et al., Multiple primary tumours: challenges and approaches, a review. *ESMO Open.* 2017;2(2):e000172 10.1136/esmoopen-2017-000172 PMC5519797 28761745

[2] Amer MH. Multiple neoplasms, single primaries, and patient survival. *Cancer Manag Res.* 2014;6:119–134 10.2147/CMAR.S57378 PMC3949559 24623992

[3] Weir HK, Johnson CJ, Thompson TD. The effect of multiple primary rules on population-based cancer survival. *Cancer Causes Control.* 2013;24(6):1231–1242 10.1007/s10552-013-0203-3 PMC4558881 23558444

[4] Slaughter DP, Southwick HW, Smejkal W. "Field cancerization" in oral stratified squamous epithelium. Clinical implications of multicentric origin. *Cancer.* 1953;6(5):963–968 10.1002/1097-0142(195309)6:5<963::aid-cncr2820060515>3.0.co;2-q 13094644

[5] Luo Y, Yu M, Grady W. Field cancerization in the colon: a role for aberrant DNA methylation?. *Gastroenterol Rep (Oxf).* 2014;2(1):16–20 10.1093/gastro/got039 PMC3920999 24760232

[6] Coyte A, Morrison DS, McLoone P. Second primary cancer risk - the impact of applying different definitions of multiple primaries: results from a retrospective population-based cancer registry study. *BMC Cancer.* 2014;14:272 10.1186/1471-2407-14-272 PMC4005906 24742063

[7] Adamo MB, Johnson CH, Ruhl JL, Dickie LA (eds.). 2012 SEER Program Coding and Staging Manual. National Cancer Institute, NIH Publication number 12–5581, Bethesda, MD

[8] Working Group Report. International rules for multiple primary cancers (ICD-0 third edition). *Eur J Cancer Prev.* 2005;14(4):307–308 10.1097/00008469-200508000-00002 16030420

[9] Jayasekara H, Reece JC, Buchanan DD, Rosty C, Ghazaleh Dashti S, Ait Ouakrim D, et al., Risk factors for metachronous colorectal cancer following a primary colorectal cancer: a prospective cohort study. *Int J Cancer.* 2016;139(5):1081–1090 10.1002/ijc.30153 PMC4911232 27098183

[10] You YN, Chua HK, Nelson H, Hassan I, Barnes SA, Harrington J. Segmental vs. extended colectomy: measurable differences in morbidity, function, and quality of life. *Dis Colon Rectum.* 2008;51(7):1036–1043 10.1007/s10350-008-9325-1 18470560

[11] Freeman HJ. Natural history and long-term outcome of patients treated for early stage colorectal cancer. *Can J Gastroenterol.* 2013;27(7):409–413 10.1155/2013/920689 PMC3956019 23862173

[12] Strate LL. Lower GI bleeding: epidemiology and diagnosis. *Gastroenterol Clin N.* 2005;34(4):643–664 10.1016/j.gtc.2005.08.007 16303575

[13] Grady E. Gastrointestinal bleeding scintigraphy in the early 21st century. *J Nucl Med.* 2015;57(2):252–259 10.2967/jnumed.115.157289 26678616

[14] Barnert J, Messmann H. Diagnosis and management of lower gastrointestinal bleeding. *Nat Rev Gastroenterol Hepatol.* 2009;6(11):637–646 10.1038/nrgastro.2009.167 19881516

[15] Abir F, Alva S, Longo WE, Audiso R, Virgo KS, Johnson FE. The postoperative surveillance of patients with colon cancer and rectal cancer. *Am J Surg.* 2006;192(1):100–108 10.1016/j.amjsurg.2006.01.053 16769285

[16] Zervos EE, Badgwell BD, Burak WE Jr, Arnold MW, Martin EW. Fluorodeoxyglucose positron emission tomography as an adjunct to carcinoembryonic antigen in the management of patients with presumed recurrent colorectal cancer and nondiagnostic radiologic workup. *Surgery.* 2001;130(4):636–644 10.1067/msy.2001.116919 11602894

[17] Hale MF. Radiation enteritis. *Curr Opin Gastroenterol.* 2020;36(3):208–214 10.1097/MOG.0000000000000632 32141897

[18] Park IJ, Yu CS, Kim HC, Jung YH, Han KR, Kim JC. Metachronous colorectal cancer. *Colorectal Dis. *2006;8(4):323–327 10.1111/j.1463-1318.2006.00949.x 16630238

[19] Center MM, Jemal A, Smith RA, Ward E. Worldwide variations in colorectal cancer. *CA Cancer J Clin.* 2009;59(6):366–378 10.3322/caac.20038 19897840

